# Workforce nurturing: an approach to improving wellbeing, burnout and professional fulfilment among Australian doctors

**DOI:** 10.5116/ijme.6639.1a23

**Published:** 2024-06-15

**Authors:** Emma Hodge, Alan Sandford

**Affiliations:** 1 Wide Bay Hospital & Health Service, Queensland, Australia

**Keywords:** Wellbeing, burnout, professional fulfilment, workforce nurturing

## Abstract

**Objective:**

To assess the workplace drivers of professional fulfilment, burnout and perceived impact of workplace issues on wellbeing in doctors working in a regional Australian hospital, following a 6-month period of comprehensive workforce nurturing strategies.

**Methods:**

An online cross-sectional survey combined both qualitative feedback and quantitative measures of wellbeing including the Stanford Professional Fulfillment Index to assess professional fulfillment and burnout and a workplace issues inventory to assess the relative perceived influence on work-related wellbeing.

**Results:**

Survey responses from 124 doctors comprised approximately 60% (n=74) prevocational doctors, 12% (n=15) registrars and 28% (n=35) specialist doctors. Around 63% (n=78) of participants were international medical graduates. Overall, 25% (n=31) reported professional fulfilment and 13% (n=13) reported burnout. The top 6 workplace issues were (i) inefficient work practices and/or processes, (ii) medical officer vacancies in my department, (iii) inadequate support staff and/or excessive admin burden, (iv) inadequate workplace staff amenities, (v) poor access to nutritious onsite food, (vi) inability to access my entitled daily meal break. Factors perceived as having a minimal impact on wellbeing included learning opportunities, rostering, access to leave and support during challenging clinical situations, were directly related to the workforce nurturing strategies implemented.

**Conclusions:**

This comprehensive evaluation of wellbeing in a regional healthcare setting provides a novel contribution to the literature by illustrating the transformative potential of workforce nurturing. Notably, the findings reflect the potential impact of workforce nurturing upon professional fulfilment and burnout, in the context of a regional hospital setting.

## Introduction

The wellbeing of healthcare professionals, especially doctors, is an emerging area of interest in research and medical leadership.^1^^-^^6^ As an occupation phenomenon, doctors experience higher levels of burnout, stress, anxiety and depression, when compared to the general population.^7^^-^^10^ Burnout occurs when workplace exhaustion, interpersonal disengagement and reduced professional efficacy ensue a period of chronic workplace stress.^11^ Suboptimal wellbeing has significant implications not only for doctors, but also for healthcare services, including high workforce turnover, medical errors, reduced productivity and patient dissatisfaction.^8^^-^^10^^,^^12^^-^^14^ In the medical context, wellbeing refers to a positive state where individuals can meet their full potential, encompassing various facets from psychosocial wellbeing to professional satisfaction.^15^^-^^16^ Professional fulfilment refers to the intrinsic positive reward derived from work which aligns with individual career aspirations.^17^^-^^18^

At the core of this is the concept of workforce nurturing, a holistic and proactive approach emphasising the cultivation of an environment which augments professional wellbeing through supportive practices, resources and organisational culture.^19^^-^^20^ More specifically, this refers to strategies which contribute to workplace improvements based on supportive environment that understands the needs of doctors. Workforce nurturing moves beyond traditional wellbeing intervention paradigms which address isolated challenges in the medical profession, such as fatigue or the unfavourable ‘resilience narrative’.^21^ Instead, it strives to create a supportive environment where doctors feel valued and empowered, emphasising the interconnectedness of personal and professional wellbeing through recognition of its intrinsic links to effective healthcare delivery.^22^^-^^24^ Central to effective workforce nurturing is a clear understanding of the challenges faced by medical professionals, especially in regional hospitals, such as resource constraints, geographical and social isolation, to feelings of professional stagnation due to limited continuous learning opportunities when compared to urban hospitals.^25^^-^^26^

Addressing such issues requires a combination of systemic reforms, targeted interventions and wellness centred leadership.^27^^-^^28^ Workplace wellbeing drivers, such as workload, work-life balance, professional relationships and opportunities for career development, are crucial components of workforce nurturing. By understanding and optimising these drivers, especially in the context of workforce shortages at regional hospitals, institutions can create an environment where doctors feel supported, valued and motivated.^29^^-^^31^

The Regional Medical Pathway (RMP), an end-to-end medical education and training pathway with a collaborative partnership between two universities and two hospital and health services, is a beacon in this regard.^32^ Through a dedicated project team, the RMP has implemented workforce nurturing through a variety of initiatives to support the wellbeing of doctors including a targeted wellbeing education program, clinical examination preparation sessions, personalised career counselling, additional pastoral care support for prevocational doctors, streamlined annual leave processes and a collaborative approach to enhancing rotational allocation preferences. This study aims to assess the professional fulfilment, burnout and perceived wellbeing following a 6-month period of this comprehensive approach to workforce nurturing. This intended to assess the efficacy of initiatives on improving the wellbeing of medical professionals and obtain a baseline measurement for future comparisons.

## Methods

### Study design

The study design was a cross-sectional survey combing a 5-point Likert scale (‘strongly agree’ to ‘strongly disagree’) of workplace drivers of wellbeing and open-ended questions. This was used to obtain a measurement of professional fulfilment and burnout, while also allowing participants to provide detail on workplace issues affecting their wellbeing. The survey also had basic demographic questions including location of primary medical degree, duration since graduation from primary medical degree, work location, department, role and duration of employment.

### Study participants

The survey was conducted at Wide Bay Hospital and Health Service (WBHHS). At the time of the survey, there were 564 doctors. All WBHHS employed doctors were eligible to participate including:

·      Prevocational doctors: those who have not yet commenced vocational specialty training including interns, resident medical officers (RMOs), principal house officers (PHOs)

·      Registrars: those on a vocational specialty training program

·      Senior medical officers (SMOs): those with vocational specialist qualifications

The survey period occurred over a 5-week period (26 June 2023 – 30 July 2023). The survey comprised a 10-minute, anonymous, voluntary questionnaire which was administered via the secure online application Microsoft Forms. All WBHHS doctors received survey invitations via email. The survey was also promoted via social media platforms, hospital intranet pages, organisation-wide posters and various educational events; during which a QR code linked to the survey was available. Informed consent was gained through acceptance of the detailed outline of the project on the first page of the survey.

### Data collection methods

The Stanford Professional Fulfillment Index (PFI) was used to provide a measure of work-related wellbeing: professional fulfillment (6-items) and burnout (10-items).^33^ This is a 16-item validated survey is designed for physicians that measures professional fulfillment (intrinsic positive reward derived from work) and burnout (work exhaustion and interpersonal disengagement). Items were scored by participants using a 5-point Likert scale (‘not at all’ to ‘completely true’ for professional fulfillment and ‘not at all’ to ‘extremely’ for burnout).

The PFI has several advantages over other more traditionally used work-related wellbeing measures such as the Maslach Burnout Index; it has a dual focus on both the positive and negative aspects of the role and work of medical officers, is relatively brief, free to reproduce and has been designed for use over time to measure the impact of interventions. This PFI is sensitivity to change and estimated test-retest reliability of 0.82 for professional fulfillment (α = 0.91), 0.80 for work exhaustion (α = 0.86), 0.71 for interpersonal disengagement (α = 0.92), and 0.80 for overall burnout (α = 0.92). Approval was received from Stanford to utilise the PFI for this project.^7^^-^^8^

In accordance with published methods, PFI items were scored from 0 to 4 and scale scores calculated by averaging the item scores within each of the domains, such that all scale scores also ranged from 0 to 4.33 A score ≥3 on the professional fulfillment scale correlated with professional fulfillment, while a score ≥1.33 on the burnout scale, correlated with burnout.

### Setting

The survey was conducted at Wide Bay Hospital and Health Service (WBHHS), a regional healthcare service with two major hospitals in Bundaberg and Hervey Bay. These facilities are located 170-400km from the closest capital city of Brisbane, Queensland.

### Data analysis

Statistical analysis was undertaken in STATA 17 by the primary author. Visual inspection of the data, descriptive statistics and the results from the Shapiro-Wilk test indicated the data is non-parametric. While the Kolmogorov-Smirnov test provided mixed results, the overall evidence indicates the data deviates from a normal distribution. Non-parametric tests were therefore used, with a significance level of p<0.05. For the open-ended questions, qualitative analysis was conducted using the Braun & Clarke method of thematic analysis.^34^

Response rates for each level of doctor were calculated using internal WBHHS data held by the medical workforce unit. Workplace wellbeing drivers were scored individually from 2 to -2, with agreement scored positively and disagreement scored negatively. Mean scores for each item were calculated to rank issues according to their perceived influence on wellbeing and the extent of agreement (both strongly agree and agree). Rates of burnout, professional fulfillment and workplace wellbeing drivers were stratified by work location, role and location of primary medical degree. All percentages are rounded to the nearest whole number.

### Ethical approval

The study protocol was submitted for ethical and scientific review and received approval from the CQHHS Human Research Ethics Committee (HREC/2022/QCQ/95343). Site specific authorisation was also sought and received from WBHHS prior to study commencement.

## Results

### Sociodemographics

A survey response was received from 124 medical officers across both Bundaberg (n=69), Hervey Bay (n=53) and Rural (n=2), representing approximately 22% of total WBHHS medical officers (n=564). Participants comprised approximately 60% (n=74) prevocational doctors, 12% (n=15) registrars and 28% (n=35) specialist doctors. A wide range of medical specialties were represented in the responses, with approximately 15 different departments. 37% (n=46) of respondents undertook their primary medical degree in Australia while 63% (n=78) completed their medical degree in countries other than Australia, which approximately reflects the proportions of the WBHHS medical workforce.

### Work-related wellbeing

Overall, 25% (n=31) reported professional fulfilment and 13% (n=16) reported burnout. 24% (n=30) of respondents reported professional fulfillment and no burnout, 12% (n=15) burnout and no professional fulfillment, <1% (n=1) reported both professional fulfillment and burnout, and 63% (n=78) met neither of the primary outcomes.

Based on the Kruskal-Wallis test, there were no statistically significant differences in professional fulfillment across work locations (p = 0.52), roles (p = 0.46), or location of primary medical degree (p = 0.83). Similarly, burnout scores remained consistent across work locations (p = 0.60) and roles (p = 0.52), with no significant variation based on the location of their primary medical degree (p = 0.09).

### Workplace wellbeing drivers

A Spearman’s correlation analysis was conducted to assess the relationship between workplace issues and participant’s perceived impact on wellbeing.

Of the 21 workplace issues, 13 issues were found to have a positive correlation as seen in Table 1, indicating overall agreement that the issue was influencing wellbeing.

**Table 1:** Workplace factors affecting wellbeing, ranked by mean Spearman’s correlation (n=124)

Workplace issues	Mean correlation (r)
Inefficient work practices and/or processes	0.73
Medical officer vacancies in my department	0.57
Inadequate support staff and/or excessive admin burden	0.44
Inadequate workplace staff amenities	0.38
Poor access to nutritious onsite food	0.38
Inability to access my entitled daily meal break	0.37
Inadequate access to resources I need to perform my role well e.g., ICT	0.18
Too few opportunities for collegial connection & fun	0.1
Inadequate recognition and/or appreciation of my effort at work	0.07
A medical culture in which overtime (rostered or unrostered) is expected and/or underpaid	0.05
Limited opportunities to attend offsite professional development	0.04
Lack of adequate sick leave cover for myself and/or my medical peers	0.03
Poorly coordinated and/or inappropriate rostering	0.01

Table 2 depicts the workplace issues with a negative Spearman’s correlation, indicating the issue did not impact wellbeing.  A Mann-Whitney U Test was utilised to examine differences in workplace issue perceptions across roles, location of primary degree and work location. A significant difference between roles was found regarding inadequate access to resources (p=0.03), inefficient work practices and/or processes also differed between roles (p= 0.04) and medical officer vacancies (p=0.05). All these issues were reported as a greater issue for specialist doctors and registrars, compared to prevocational doctors.

**Table 2:** Workplace factors not affecting wellbeing, ranked by mean Spearman’s correlation (n=124)

Workplace Issue	Mean correlation (r)
Lack of onsite quality learning opportunities	-0.02
Inflexible rostering e.g. limited opportunities for part time work or shift preferencing	-0.07
A lack of workplace fairness and/or transparency	-0.12
Workplace unprofessionalism e.g. bullying, discrimination, harassment	-0.37
Inability to access annual leave when I want it	-0.38
Lack of support when I face challenging clinical situations e.g. a clinical incident, patient death, patient complaint	-0.4
Insufficient control and/or autonomy in my work (appropriate to my scope of practice)	-0.45
Misalignment of my current role with my future career goals	-0.46

Based on location of primary medical degree, Australian medical graduates (AMGs) perceive inadequate access to resources (p=0.04), inefficient work practices and/or processes (p=0.02), lack of onsite quality learning opportunities (p=0.05) and medical officer vacancies (p=0.03) as a more significant concern, when compared to International medical graduates (IMGs).

A Mann-Whitney U Test was also employed to compare scores between Bundaberg & Rurals and Hervey Bay. With regards to inadequate support staff and/or excessive admin burden (p=0.03), inefficient work practices and/or processes (p-value= 0.00) and inadequate recognition and/or appreciation of my effort at work (p-value= 0.01), these were perceived as a more significant concern for Bundaberg & Rurals, when compared to Hervey Bay.

### Open-ended survey responses

The emerging themes within the free text responses related to staffing shortages, inefficient work practices as well as feedback and training. Workforce configuration, even in fully staffed departments, was a pervasive concern among many departments, particularly relating to the numbers of prevocational doctors. Some concerns were raised about a perceived disconnect from executive leadership and the apparent unwillingness to address key issues, such as staffing shortages.

The need for improved information technology infrastructure, including an electronic medical record system, was strongly emphasised. Suggestions for process improvements, such adopting electronic processes for imaging requests as opposed to dropping off paper forms, were brought up as possible strategies to enhance efficiency. Timely access to rosters and more flexible rostering options were also frequently mentioned as recurring issues. The availability of hospital beds and addressing bed block issues were also raised. A desire for more specific and targeted feedback from clinical supervisors and opportunities for bedside teaching was expressed. Some prevocational doctors outlined their concerns about barriers to training programs and professional development. A conducive environment, including a common area for junior doctors, was highlighted as a crucial need to allow breaks and improve morale. Respondents indicated they often faced barriers in accessing their entitled meal break and the ability to leave work on time.

## Discussion

The intricate relationship between workforce nurturing and the outcomes on professional fulfillment, burnout and workplace wellbeing drivers emerged prominently in this study. The proportion of doctors with professional fulfilment aligns with prior literature findings, although research largely examines professional fulfilment in doctors with specialist registration.^35^^-^^38^ Rates of burnout reported in this survey were notably lower than national and international averages, with rates previously documented between 30-40%.^35^^-^^47^ The uniformity in perceptions of professional fulfillment and burnout across different factors, such as work locations and role level, indicates the common workplace challenges remain consistent across various backgrounds and settings.

While respondents still conveyed their challenges with fatigue and staffing shortages, this favourable comparison emphasises the influence of workforce nurturing. The literature reflects that while burnout is a multifaceted issue, a nurturing environment which addresses both systemic issues and intangible factors can significantly mitigate its effects.^48^^,^^49^

Workplace issues shown to impact the wellbeing of participants centred around workplace inefficiencies and an extensive workload relative to staffing numbers. These were seen as potential barriers to access entitled daily meal breaks, due to the volume of work, burden of administrative tasks and the ensuing culture of expected overtime. This highlights the need to identify opportunities for work practice reform to streamline efficiency, such as enhancing technological infrastructure.^50^^, ^^51^ Feedback also reflected an overarching imperative for further local opportunities to pursue accredited specialist medical training, a known challenge for regional healthcare settings.^9^

The negative correlations with certain wellbeing drivers observed in the present study suggest the prior interventions implemented at this regional hospital, informed by the principles of workforce nurturing, have already started to show positive effects. Such initiatives have included a dedicated wellbeing education program, clinical examination preparation sessions, personalised career counselling, additional pastoral care support for prevocational doctors, streamlined annual leave processes and a collaborative approach to enhancing rotational allocation preferences. Underpinning these interventions is a strong emphasis on a supportive culture with continuous rapport building, through the novel innovation of a dedicated medical education and wellbeing position. A sense of control and autonomy emerged as one such driver, a concept which has been spotlighted in the literature as being pivotal in professional settings, as doctors who perceive influence over their schedules often report diminished burnout and increased job satisfaction.^52^ This reaffirms the notion a nurturing environment can actively foster a sense of autonomy and control, thereby enhancing wellbeing. Similarly, the alignment of doctors’ current roles with future career aspirations showcases the strong focus on supporting career progression at this regional hospital. Such alignment plays a pivotal role in career wellbeing and workforce sustainability.^53^

Moreover, modifications to rostering and annual leave processes, as well as expansions to onsite education are further evident as factors driving wellbeing in this cohort of doctors. Positive feedback regarding the degree of support during challenging clinical situations highlights the benefits of a strong emphasis on wellbeing and rapport building, particular for prevocational doctors. These findings further elucidate the relationship between workforce nurturing and professional fulfillment. When doctors work within a supportive and growth-oriented environment, they are more inclined to find profound satisfaction in their roles.^54^ This posits the role of nurturing as both a preventive measure against adverse outcomes such as burnout, as well as a proactive measure to foster positives like professional fulfillment.^55^^, ^^56^

Moreover, the implementation of clear and robust mechanisms to address any concerns of bullying or harassment in the workplace, as well as collaborative mentorship programs, were reflected in findings related to workplace unprofessionalism. Creating an environment which prioritises the mental and emotional wellbeing of staff is a cornerstone of the principles of workforce nurturing.^57^

The challenges unique to regional hospitals, such as resource limitations and geographical and social isolation, magnify the implications of the study's findings.^58^ Despite these constraints, a steadfast commitment to workforce nurturing can induce positive outcomes in professional wellbeing. This insight holds significant ramifications for regional healthcare policy and strategy, suggesting workforce nurturing may be pivotal in initiatives aimed at attracting and retaining doctors in regional settings.^59^

The main limitation of this research relates to the sample size, with certain experiences or attitudes potentially influencing the proclivity to respond to the survey. Informal feedback throughout the survey period indicated a consensus of survey fatigue, as medical officers are routinely inundated with requests to complete a plethora of questionnaires.^58^ Nevertheless, considering these practical constraints, data has been collected from a reasonable number of doctors across each role level, department and work location in the sample population. Additionally, there was no baseline measurement of wellbeing for this regional hospital which prevented a post-intervention comparison.

Overall, this study highlights the multifaceted challenges faced by medical professionals and suggests workforce nurturing could indeed be the linchpin in resolving a myriad of workplace wellbeing drivers, with positive influences upon burnout and professional fulfillment. As healthcare systems worldwide grapple with professional wellbeing challenges, this study offers an exemplary blueprint for transformative change anchored in workforce nurturing principles.

## Conclusions

This study has illustrated the profound impact of creating a nurturing environment in optimising the workplace experience of medical professionals, especially in the challenging milieu of regional healthcare. Despite inherent challenges in regional healthcare, the relatively low rates of burnout and consistent rates of professional fulfillment highlight the powerful impact of recent interventions at this regional hospital. Such findings provide a compelling blueprint for medical educators and teaching hospitals while highlighting the transformative potential of a nurturing environment to meaningfully mitigate burnout, enhance professional fulfillment, foster a sustainable medical workforce and ultimately enhance patient care delivery. Future studies should explore the impact of different strategies which underpin this novel concept of workforce nurturing. As the landscape of medical education continues to evolve, workforce nurturing is a crucial consideration in ensuring the sustainability, growth and wellbeing of medical professionals.

### Conflicts of Interest

The authors declare they have no conflicts of interest.

## References

[1] Sinskey JL, Margolis RD, Vinson AE (2022). The wicked problem of physician well-being... Anesthesiol Clin.

[2] Shanafelt TD. Enhancing meaning in work: a prescription for preventing physician burnout and promoting patient-centered care. JAMA. 2009; 302(12):1338-40. 10.1001/jama.2009.1385 19773573

[3] West CP, Dyrbye LN, Erwin PJ, Shanafelt TD (2016). Interventions to prevent and reduce physician burnout: a systematic review and meta-analysis.. Lancet.

[4] Eckleberry-Hunt J, Lick D, Boura J, Hunt R, Balasubramaniam M, Mulhem E, Fisher C (2009). An exploratory study of resident burnout and wellness.. Acad Med.

[5] Shanafelt TD, Noseworthy JH (2017). Executive leadership and physician well-being: nine organizational strategies to promote engagement and reduce burnout.. Mayo Clin Proc.

[6] Shanafelt T, Trockel M, Ripp J, Murphy ML, Sandborg C, Bohman B (2019). Building a program on well-being: key design considerations to meet the unique needs of each organization.. Acad Med.

[7] Beyondblue. National mental health survey of doctors and medical stu-dents. 2013. [Cited 22 March 2024]; Available from: https://medicine.uq.edu.au/files/42088/Beyondblue%20Doctors%20Mental%20health.pdf.

[8] Parr JM, Pinto N, Hanson M, Meehan A, Moore PT. Medical graduates, tertiary hospitals, and burnout: a longitudinal cohort study. Ochsner J. 2016;16(1):22-6. PMC4795494 27046399

[9] Clough BA, Ireland MJ, Leane S, March S (2020). Stressors and protective factors among regional and metropolitan Australian medical doctors: a mixed methods investigation.. J Clin Psychol.

[10] Shanafelt TD, Mungo M, Schmitgen J, Storz KA, Reeves D, Hayes SN, Sloan JA, Swensen SJ, Buskirk SJ (2016). Longitudinal study evaluating the association between physician burnout and changes in professional work effort.. Mayo Clin Proc.

[11] World Health Organisation. Burn-out an "occupational phenomenon". 2019. [Cited 22 March 2024]; Available from: https://www.who.int/standards/classifications/frequently-asked-questions/burn-out-an-occupational-phenomenon.

[12] Sterling R, Rinne ST, Reddy A, Moldestad M, Kaboli P, Helfrich CD, Henrikson NB, Nelson KM, Kaminetzky C, Wong ES (2022). Identifying and prioritizing workplace climate predictors of burnout among vha primary care physicians.. J Gen Intern Med.

[13] Dewa CS, Jacobs P, Thanh NX, Loong D (2014). An estimate of the cost of burnout on early retirement and reduction in clinical hours of practicing physicians in Canada.. BMC Health Serv Res.

[14] Ryan E, Hore K, Power J, Jackson T (2023). The relationship between physician burnout and depression, anxiety, suicidality and substance abuse: a mixed methods systematic review.. Front Public Health.

[15] Simons G, Baldwin DS (2021). A critical review of the definition of 'wellbeing' for doctors and their patients in a post Covid-19 era.. Int J Soc Psychiatry.

[16] Raj KS (2016). Well-being in residency: a systematic review.. J Grad Med Educ.

[17] Oliveira-Silva LC, Porto JB. Subjective well-being and flourishing at work: the impact of professional fulfilment. RAM Rev Administração Mackenzie. 2021;22.

[18] Prins JT, Gazendam-Donofrio SM, Tubben BJ, van der Heijden FM, van de Wiel HB, Hoekstra-Weebers JE (2007). Burnout in medical residents: a review.. Med Educ.

[19] Levkovich N (2016). The fourth aim: How do we care for our healthcare workforce?. Fam Syst Health.

[20] Andersson T, Linnéusson G, Holmén M, Kjellsdotter A (2023). Nurturing innovative culture in a healthcare organisation - lessons from a Swedish case study.. J Health Organ Manag.

[21] Hodge E, Witter E. Doctors-in-training need system reform, not more resilience. 2023. [Cited 22 March 2024]; Available from: https://insightplus.mja.com.au/2023/6/doctors-in-training-need-system-reform-not-more-resilience/.

[22] Shanafelt TD, Gorringe G, Menaker R, Storz KA, Reeves D, Buskirk SJ, Sloan JA, Swensen SJ (2015). Impact of organizational leadership on physician burnout and satisfaction.. Mayo Clin Proc.

[23] Lemaire JB, Wallace JE (2010). Not all coping strategies are created equal: a mixed methods study exploring physicians' self reported coping strategies.. BMC Health Serv Res.

[24] Yoo PS, Tackett JJ, Maxfield MW, Fisher R, Huot SJ, Longo WE (2017). Personal and professional well-being of surgical residents in new England.. J Am Coll Surg.

[25] Keane S, Lincoln M, Smith T (2012). Retention of allied health professionals in rural New South Wales: a thematic analysis of focus group discussions.. BMC Health Serv Res.

[26] Lu DW, Lee J, Alvarez A, Sakamoto JT, Bird SB, Sundaram V, Lall MD, Nordenholz KE, Manfredi RA, Blomkalns AL (2022). Drivers of professional fulfillment and burnout among emergency medicine faculty: a national wellness survey by the Society for academic emergency medicine.. Acad Emerg Med.

[27] Shanafelt T, Trockel M, Rodriguez A, Logan D (2021). Wellness-centered leadership: equipping health care leaders to cultivate physician well-being and professional fulfillment.. Acad Med.

[28] Burns KEA, Pattani R, Lorens E, Straus SE, Hawker GA (2021). The impact of organizational culture on professional fulfillment and burnout in an academic department of medicine.. PLoS One.

[29] Panagioti M, Panagopoulou E, Bower P, Lewith G, Kontopantelis E, Chew-Graham C, Dawson S, van Marwijk H, Geraghty K, Esmail A (2017). Controlled interventions to reduce burnout in physicians: a systematic review and meta-analysis.. JAMA Intern Med.

[30] Dolea C, Adams O. Motivation of health care workers-review of theories and empirical evidence. Cah Sociol Demogr Med. 2005;45(1):135-61. 15938446

[31] Firth-Cozens J (2001). Interventions to improve physicians' well-being and patient care.. Soc Sci Med.

[32] University of Queensland. Regional Medical Pathway. 2021. [Cited 22 March 2024]; Available from: https://stories.uq.edu.au/medicine/2021/regional-medical-pathway-offers-new-career-choices-closer-to-home/index.html.

[33] Trockel M, Bohman B, Lesure E, Hamidi MS, Welle D, Roberts L, Shanafelt T (2018). A brief instrument to assess both burnout and professional fulfillment in physicians: reliability and validity, including correlation with self-reported medical errors, in a sample of resident and practicing physicians.. Acad Psychiatry.

[34] Braun V, Clarke V (2014). What can "thematic analysis" offer health and wellbeing researchers?. Int J Qual Stud Health Well-being.

[35] Sakamoto JT, Lee J, Lu DW, Sundaram V, Bird SB, Blomkalns AL, Alvarez A (2022). Factors driving burnout and professional fulfillment among emergency medicine residents: a national wellness survey by the Society for Academic Emergency Medicine.. AEM Educ Train.

[36] Karras R, Matos S, Sharma A, Crosby DL (2022). Predictors of professional fulfillment and burnout among otolaryngologists during the COVID-19 pandemic.. OTO Open.

[37] Makowski MS, Trockel M, Paganoni S, Weinstein S, Verduzco-Gutierrez M, Kinney C, Kennedy DJ, Sliwa J, Wang H, Knowlton T, Stautzenbach T, Shanafelt TD (2023). Occupational characteristics associated with professional fulfillment and burnout among US physiatrists.. Am J Phys Med Rehabil.

[38] Lazarides AL, Belay ES, Anastasio AT, Cook CE, Anakwenze OA (2021). Physician burnout and professional satisfaction in orthopedic surgeons during the COVID-19 Pandemic.. Work.

[39] Parr JM, Pinto N, Hanson M, Meehan A, Moore PT. Medical graduates, tertiary hospitals, and burnout: a longitudinal cohort study. Ochsner J. 2016;16(1):22-6. PMC4795494 27046399

[40] Raftopulos M, Wong EH, Stewart TE, Boustred RN, Harvey RJ, Sacks R (2019). Occupational burnout among otolaryngology-head and neck surgery trainees in Australia.. Otolaryngol Head Neck Surg.

[41] Chambers CN, Frampton CM, Barclay M, McKee M (2016). Burnout prevalence in New Zealand's public hospital senior medical workforce: a cross-sectional mixed methods study.. BMJ Open.

[42] Hamidi MS, Bohman B, Sandborg C, Smith-Coggins R, de Vries P, Albert MS, Murphy ML, Welle D, Trockel MT (2018). Estimating institutional physician turnover attributable to self-reported burnout and associated financial burden: a case study.. BMC Health Serv Res.

[43] Windover AK, Martinez K, Mercer MB, Neuendorf K, Boissy A, Rothberg MB (2018). Correlates and outcomes of physician burnout within a large academic medical center.. JAMA Intern Med.

[44] Nori P, Bartash R, Cowman K, Dackis M, Pirofski LA (2019). Is burnout infectious? understanding drivers of burnout and job satisfaction among academic infectious diseases physicians.. Open Forum Infect Dis.

[45] Mir H, Downes K, Chen AF, Grewal R, Kelly DM, Lee MJ, Leucht P, Dulai SK (2021). Physician wellness in orthopaedic surgery : a multinational survey study.. Bone Jt Open.

[46] Dobson H, Malpas CB, Burrell AJ, Gurvich C, Chen L, Kulkarni J, Winton-Brown T (2021). Burnout and psychological distress amongst Australian healthcare workers during the COVID-19 pandemic.. Australas Psychiatry.

[47] Maslach C, Leiter MP (2016). Understanding the burnout experience: recent research and its implications for psychiatry.. World Psychiatry.

[48] West CP, Dyrbye LN, Shanafelt TD (2018). Physician burnout: contributors, consequences and solutions.. J Intern Med.

[49] Ferguson C, Low G, Shiau G (2020). Resident physician burnout: insights from a Canadian multispecialty survey.. Postgrad Med J.

[50] Agarwal SD, Pabo E, Rozenblum R, Sherritt KM (2020). Professional dissonance and burnout in primary care: a qualitative study.. JAMA Intern Med.

[51] Chaudhry B, Wang J, Wu S, Maglione M, Mojica W, Roth E, Morton SC, Shekelle PG (2006). Systematic review: impact of health information technology on quality, efficiency, and costs of medical care.. Ann Intern Med.

[52] Hartzband P, Groopman J (2020). Physician burnout, interrupted.. N Engl J Med.

[53] Wilhelm F, Hirschi A. Career self-management as a key factor for career well-being. In: Potgieter IL, Ferreira N, Coetzee, editors. Theory, research, and dynamics of career well-being: becoming fit for the future. Gewerbestrasse: Springer; 2019.

[54] Handoyo NE, Claramita M, Keraf MKPA, Ash J, Schuwirth L, Rahayu GR (2023). The importance of developing meaningfulness and manageability for resilience in rural doctors.. Med Teach.

[55] de Koning R, Egiz A, Kotecha J, Ciuculete AC, Ooi SZY, Bankole NDA, Erhabor J, Higginbotham G, Khan M, Dalle DU, Sichimba D, Bandyopadhyay S, Kanmounye US (2021). Survey fatigue during the COVID-19 pandemic: an analysis of neurosurgery survey response rates.. Front Surg.

[56] Gadolin C, Andersson T, Eriksson E, Hellström A (2020). Providing healthcare through “value shops”: impact on professional fulfilment for physicians and nurses.. International Journal of Health Governance.

[57] Cohen C, Pignata S, Bezak E, Tie M, Childs J (2023). Workplace interventions to improve well-being and reduce burnout for nurses, physicians and allied healthcare professionals: a systematic review.. BMJ Open.

[58] Armstrong BK, Gillespie JA, Leeder SR, Rubin GL, Russell LM (2007). Challenges in health and health care for Australia.. Med J Aust.

[59] Bourke L, Waite C, Wright J (2014). Mentoring as a retention strategy to sustain the rural and remote health workforce.. Aust J Rural Health.

